# Dual β-lactams for the treatment of *Mycobacterium abscessus*: a review of the evidence and a call to act against an antibiotic nightmare

**DOI:** 10.1093/jac/dkae288

**Published:** 2024-08-16

**Authors:** Bianca Maria Longo, Mattia Trunfio, Andrea Calcagno

**Affiliations:** Department of Medical Sciences, Unit of Infectious Diseases, Amedeo di Savoia Hospital, University of Turin, 10149 Turin, Italy; Department of Medical Sciences, Unit of Infectious Diseases, Amedeo di Savoia Hospital, University of Turin, 10149 Turin, Italy; Department of Medicine, Division of Infectious Diseases and Global Public Health, University of California San Diego, La Jolla, CA 92037, USA; Department of Medical Sciences, Unit of Infectious Diseases, Amedeo di Savoia Hospital, University of Turin, 10149 Turin, Italy

## Abstract

*Mycobacterium abscessus* complex is a group of rapidly growing non-tuberculous mycobacteria (NTM), increasingly emerging as opportunistic pathogens. Current treatment options for these microorganisms are limited and associated with a high rate of treatment failure, toxicity and recurrence. In search of new therapeutic strategies, interest has grown in dual β-lactam (DBL) therapy, as research recently discovered that *M. abscessus* cell wall synthesis is mainly regulated by two types of enzymes (d,d-transpeptidases and l,d-transpeptidases) differently susceptible to inhibition by distinct β-lactams. *In vitro* studies testing several DBL combinations have shown synergy in extracellular broth cultures as well as in the intracellular setting: cefoxitin/imipenem, ceftaroline/imipenem, ceftazidime/ceftaroline and ceftazidime/imipenem. The addition of specific β-lactamase inhibitors (BLIs) targeting *M. abscessus* β-lactamase did not significantly enhance the activity of DBL combinations. However, *in vivo* data are lacking. We reviewed the literature on DBL/DBL-BLI-based therapies for *M. abscessus* infections to raise greater attention on this promising yet overlooked treatment option and to guide future preclinical and clinical studies.

## Introduction

*Mycobacterium abscessus* complex is a group of rapidly growing non-tuberculous mycobacteria (NTM), ubiquitous in the environment, and increasingly emerging as opportunistic pathogens.^1^ Although mainly environmentally acquired, recent WGS analysis targeting *M. abscessus* strains has suggested the possibility of human-to-human transmission.^2^  *M. abscessus* causes a wide range of clinical diseases such as pulmonary, skin and soft tissue infections, especially in the setting of immunosuppression, pre-existing lung diseases and as post-surgical or post-traumatic complications.^3^ Disseminated disease and osteoarticular involvement have also been documented in the literature.^4,5^ Frequently, the infection has poor outcomes, with cure rates as low as 30%.^6,7^ This is especially true in the setting of cystic fibrosis, where lung function decline has been demonstrated to correlate with *M. abscessus* detection.^8,9^

Management of *M. abscessus* is extremely challenging for several reasons, including the ability of *M. abscessus* to thrive in hostile environments thanks to biofilm production^10,11^ and reduced susceptibility to disinfectants,^12^ as well as the tendency to develop multidrug resistance.^13,14^  *M. abscessus* displays both intrinsic and acquired resistance to multiple antibiotics, thereby limiting therapeutic options and demanding combination therapy.^15,16^ Furthermore, antibiotic susceptibility varies across the three subspecies of *M. abscessus* complex recently identified (subsp. *massiliense*, subsp. *abscessus* and subsp. *bolletii*), with the latter two harbouring a functional *erm*(41) gene, which confers inducible macrolide resistance.^17,18^

Current guidelines for the treatment of *M. abscessus* infections recommend combination regimens that require at least three to four active drugs selected according to *in vitro* susceptibility^19^ (common recommended regimens are reported in Table 1). Due to the paucity of clinical trials, these recommendations are based on low-strength evidence. Eventually, despite prolonged ‘targeted’ regimens, treatment failures, recurrence and chronic infections are common, alongside high rates of treatment discontinuation and loss to follow-up due to drug-related toxicity.^6^ In 2012, *M. abscessus* was labelled as an ‘antibiotic nightmare’^21^ and no consistent improvement has been achieved over the last decade.

**Table 1.** ATS/ERS/ESCMID/IDSA Clinical Practice Guideline: recommended treatment for *M. absessus*^19^

Preferred drugs^a^	Number of drugs	Dosing scheme	Duration
Initial phase * *Parenteral Amikacin Imipenem (or cefoxitin) Tigecycline * *Oral Azithromycin (clarithromycin)**^b^** Clofazimine Linezolid	≥ 3 for macrolide-susceptible *M. abscessus* ≥ 4 for macrolide inducible or mutational resistant *M. abscessus*	Daily (3 times weekly may be used for aminoglycosides)	On average >12 months**^c^**
Continuation phase * *Oral/inhaled Azithromycin (clarithromycin)**^b^** Clofazimine Linezolid Inhaled amikacin	2 for macrolide-susceptible *M. abscessus* ≥ 3 for macrolide inducible or mutational resistant *M. abscessus*	Daily (3 times weekly may be used for aminoglycosides)	On average >12 months**^c^**

^a^The choice should be guided by *in vitro* susceptibility.

^b^In the case of inducible or mutational macrolide resistance, macrolides can be added for their immunomodulatory effects, but do not count as active drugs.

^c^Expert consultation should be obtained prior to initiation of therapy to determine whether a shorter or longer treatment regimen should be used. Among the factors that should guide treatment duration, the site of infection, the type of lesions (e.g. cavitary versus nodular/bronchiectatic), *M. abscessus* subspecies (e.g. potentially shorter for subsp. *massiliense*), drug susceptibility and toxicity, and opportunity of surgical resection. Overall, most of the published studies on pulmonary infection included participants treated for a minimum of 2–4 weeks with parenteral therapy, followed by oral therapy for at least 12 months from sputum conversion. Shorter oral regimens have been attempted for soft-tissue/skin infections and isolated bacteraemia, but no consensus has been met.^20^

In response to the need for enhanced therapeutic approaches, the interest in additional, safe and effective antibiotics is growing. Among the candidates, dual β-lactam (DBL) therapy against *M. abscessus* has been investigated *in vitro* and *in vivo*, and yet its acknowledgement as an additional weapon in the antibiotic armamentarium against *M. abscessus* falls behind. Despite the safety profile and the preliminary evidence of efficacy,^22^ this class of antibiotics has been understudied in the case of *M. abscessus*, with most of the data coming from research on *Mycobacterium tuberculosis*. Currently, only cefoxitin and imipenem are considered in the guidelines for this NTM.^19^

Among the reasons for scepticism are the considerable variability of susceptibility among *M. abscessus* clinical isolates, which complicates the selection of therapeutic regimens in clinical practice,^23^ the *M. abscessus* thick cell wall, rich in hydrophobic molecules that could restrict antibiotic penetration,^24–26^ and the production of a chromosomally encoded β-lactamase, Bla_Mab_.^27^

However, recent insights into the unique interaction between β-lactams and their enzymatic targets in *M. abscessus* have been described. MICs of β-lactams for different strains of *M. abscessus* have been recently characterized,^28–30^ and various DBL combinations have shown *in vitro* synergy against clinical isolates.^31,32^ As elucidated by other models of infections, such as for *Enterococcus faecalis*,^33^ the rationale for combining two β-lactams stems from the fact that the members of this class can target distinct enzymes involved in different steps of the synthesis of the bacterial cell wall.

In this narrative review we reviewed and analysed the available literature reporting on DBL therapy for *M. abscessus* with the aim of shedding light on a potential yet underexplored treatment option. Our search strategy included only English-written publications retrieved from PubMed, MEDLINE and Embase through the following Mesh terms: ‘*beta-lactams*’ OR ‘*dual beta-lactam therapy*’ OR ‘*double beta-lactam therapy*’ OR ‘*beta-lactams combinations*’ OR ‘*beta-lactam synergism*’ OR ‘*double carbapenem*’ OR ‘*beta-lactamase*’ OR ‘*beta-lactamase inhibitor*’ OR ‘*avibactam*’ AND either ‘*Mycobacterium abscessus*’ OR ‘*nontuberculous mycobacterium*’. Only manuscripts published from January 2010 to January 2023 were included.

## Transpeptidases of *M. abscessus*: the pharmacological rationale for DBLs

Peptidoglycan synthesis in *M. abscessus* is mainly regulated by two types of enzymes: the canonical d,d-transpeptidases (DDTs), also known as high-molecular-weight PBPs and the primary target of β-lactams,^34^ and the newly discovered l,d-transpeptidases (LDTs).^35^ LDTs differ from DDTs in their capacity to generate 3′-3′ linkages between oligopeptides of the bacterial cell wall, rather than 4′-3′ linkages,^36,37^ and unlike most bacteria, the activity of LDTs predominates in mycobacteria, including *M. abscessus.*^37^ Additionally implied in *M. abscessus* cell wall formation is a d,d-carboxypeptidase (DDC), a low-molecular-weight PBP, which is also targeted by different β-lactams.^38–41^ Considering all of this, the presence of several targets for β-lactams, all essential in *M. abscessus*, represents a strong argument in support of the possibility of using DBLs to achieve synergistic effects; in fact, these classes of enzymes are also differently susceptible to inhibition by distinct β-lactams.^42^

As for *M. tuberculosis*,^43^ five different transpeptidases—Ldt_Mab1–5_—are encoded in the genome of *M. abscessus*,^44,45^ each exhibiting structural and functional peculiarities. The primary sequence of these five LDTs displays considerable variability, which may account for the different binding preferences within β-lactams. Sequence identity among Ldt_Mab1–5_ is as low as 9%, with an overall similarity of about 25%, which reaches up to about 70% between Ldt_Mab1_, Ldt_Mab2_ and Ldt_Mab4_, and between Ldt_Mab3_ and Ldt_Mab5_.^38^

**Figure 1.**
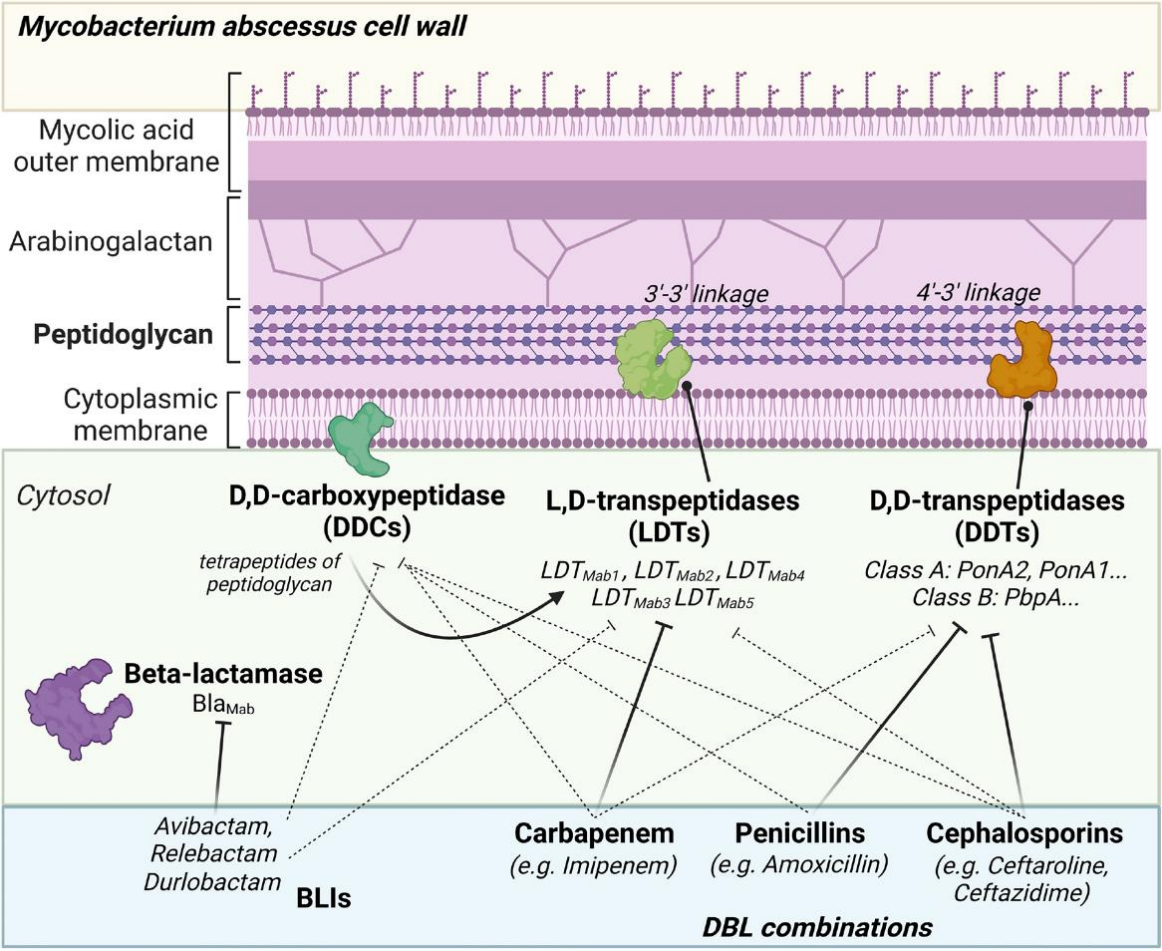
Interactions between *M. abscessus* cell wall transpeptidases (DDCs, LDTS and DDTs), Bla_Mab_ and β-lactams/BLIs. Lines indicate proven interaction between target enzymes and β-lactams/BLIs (continuous lines for more robust evidence; dotted lines for weaker evidence). Absence of a line indicates no demonstrated interaction. Within each β-lactam subclass, molecules with a greater abundance of reported data in the literature were highlighted as example. This figure appears in colour in the online version of *JAC* and in black and white in the print version of *JAC*.

In line with this structural heterogeneity, diverse β-lactams have demonstrated varying levels of relative inhibition of *M. abscessus* LDTs (see Figure 1). In fact, LDTs in *M. abscessus* are preferentially targeted by carbapenems and, to a lesser extent, by cephalosporins, with differences in terms of relative inhibition within the same antibiotic subclasses.^23,42^ On the contrary, penicillins have shown limited or no effect on these enzymes.^38,42^ Some β-lactamase inhibitors (BLIs), such as relebactam and avibactam, have also been found to create covalent bonds with LDTs.^40,46,47^ It is therefore conceivable that in addition to inhibiting Bla_Mab_, they could interfere with peptidoglycan synthesis, directly targeting functionally formed transpeptidases, as previously described in the context of other bacteria, such as Enterobacteriaceae.^48–50^

Further data are warranted to clarify the spectrum of interactions, targets and synergy mechanisms among the class of β-lactams for *M. abscessus*, as evidence has also reported findings that differ from what can be postulated and expected based on previous DBL models. As an example, Dousa *et al*.^38^ investigated the action of ceftaroline and imipenem on *M. abscessus* LDTs. When coincubated, this combination showed synergistic effects against *M. abscessus*;^38^ however, both bind to the same enzymes (Ldt_Mab1,2,4,5_) and neither bind to Ldt_Mab3_, and when coincubated imipenem was identified as the preferential substrate of LDTs due to its greater affinity.^38^ This unexpected target redundancy may suggest competitive antagonism rather than synergism between the two drugs.^38^ Considering other models of DBL therapy, we are more acquainted with the concept that synergism takes place through the concurrent inhibition of different transpeptidases in a complementary manner (e.g. for *E. faecalis*, ceftriaxone targets PBP2 and PBP3, while ampicillin inhibits PBP4 and PBP5).^33^ The authors provided two possible and non-mutually exclusive explanations, supported by further *in silico* analyses conducted on Ldt_Mab2_:^51^ first, a quantitative effect caused by the sum of both drugs upon the same target, and second, the possibility that the preferential binding of imipenem to Ldt_Mab2_ could trigger a morphological change in the target enzyme, thereby favouring the binding of ceftaroline, which itself would have less affinity (ligand-induced conformational change).^38,51^

Overall, there is a relative paucity of descriptive data on the structural and binding properties for *M. abscessus* DDTs that generate 4′-3′ linkages. Sayed *et al*.^39^ identified several DDTs with differing expression levels in *M. abscessus*. Among them, two class A PBPs (PonA2, PonA1) and one class B PBP (PbpA) were more comprehensively characterized via proteomics.^39^ The first two are bifunctional PBPs, serving both as transglycosylases and as transpeptidases, while PbpA is a monofunctional DDT. Their interaction with 13 β-lactams and 2 BLIs was investigated and quantified as half maximal inhibitory concentration (IC_50_). Carbapenems, ceftriaxone and cefotaxime effectively inhibited all these PBPs at concentrations lower than those usually reached in humans for clinical purpose. Ceftazidime and cefoxitin required higher concentrations. The remaining drugs, including penicillins (e.g. ampicillin) showed unsatisfactory results.^39^

Within the PBP family expressed in *M. abscessus*, a DDC has also been identified to catalyse the hydrolysis of the terminal end of peptide stems.^38,39^ Interestingly, this DDC is crucial for the function of LDTs, because of its ability to convert pentapeptides of peptidoglycan subunits to tetrapeptides, which are the preferential substrates of LDTs.^41^ In recent analyses investigating its interaction with β-lactams, *M. abscessus* DDC was able to strongly bind to imipenem and, to a lesser extent, to ceftaroline and avibactam.^38^ Additionally, unlike LDTs, which lack affinity for penicillins, DDC was also able to stably bind to amoxicillin.^40^

Overall, carbapenems, followed by cephalosporins, seem to be the most effective class among β-lactams to inhibit *M. abscessus* transpeptidases, with due differences within subclasses. The interplay between β-lactams and *M. abscessus* target enzymes is further complicated by the functional and structural heterogeneity existing among different transpeptidase subtypes, with paucity of data and limited possibilities of translating information from other better detailed models of infection. These findings underline how the mechanisms by which DBL-based strategies can overcome antibiotic resistance or enhance the therapeutic effectiveness against *M. abscessus* may differ compared with those already detailed for other bacteria and represent a call to action for acquiring further data in the field.

## DBL strategy in *M. abscessus*

### In vitro studies

#### DBL combinations

Ten studies assessing the effects of DBL therapy against *M. abscessus* have been published at the time of writing this review^31,32,38,41,42,52–56^ (Table 2). Most of the studies (80%) on DBL combined cephalosporins and carbapenems with or without BLIs. All of them used ATCC 19977 as the reference *M. abscessus* strain and seven studies also tested DBL efficacy against *M. abscessus* clinical isolates. MICs were determined through susceptibility testing using broth microdilution assays, and synergy was assessed through chequerboard assays, allowing for calculation of an FIC index (FICI) for each drug. The FICI is a mathematical representation of the degree to which each drug in a combination contributes to synergy of the pair against each isolate (FICIs of ≤0.5 indicating synergy, FICIs of >0.5 to <4 indifference, and FICIs of >4 antagonism).^57,58^ Two studies additionally examined the bactericidal and intracellular activities of β-lactam combinations using time–kill assays and THP-1 macrophage assays.^41,53^ One study was based on a murine model,^52^ while the remaining were *in vitro* studies.

**Table 2.** Summary of the *in vitro* and in-animal model studies on DBL for *M. abscessus*

Study	Methods	Bacterial strains	Tested combinations	Main results
Story-Roller *et al.*, 2019^31^	*In vitro* synergy AST by chequerboard titration assay (72 h incubation at 30°C). Calculation of frequency of spontaneous drug resistance selection in the presence of drug combinations (7 day incubation at 37°C).	ATCC 19977	206 drug combinations: cephalosporins + carbapenems/penem^a^, a rifamycin + either a cephalosporin or carbapenem/penem^a^, and avibactam or clavulanate + cephalosporin or carbapenem/penem^a^	12/206 Combinations were synergistic and exhibited MICs below presumed breakpoints for both single drugs: biapenem/avibactam, cefoxitin/imipenem, imipenem/doripenem, cefdinir/imipenem, imipenem/biapenem, cefuroxime/avibactam, cefditoren/imipenem, cefuroxime/cefditoren, cefditoren/biapenem, cefuroxime/imipenem, cefpodoxime/imipenem, imipenem/avibactam. The synergistic combinations showed lower resistant mutant selection frequency than each drug individually.
Story-Roller *et al.*, 2021^32^	*In vitro* synergy AST by chequerboard titration assay (72 h incubation at 30°C).	21 clinical respiratory isolates from CF patients	13 drug combinations: cefoxitin/imipenem, cefuroxime/imipenem, doripenem/imipenem, biapenem/imipenem, cefdinir/imipenem, faropenem/imipenem, cefadroxil/tebipenem, ertapenem/imipenem, cefditoren/imipenem, cefpodoxime/imipenem, cefditoren/tebipenem, cefuroxime/cefditoren, cefditoren/biapenem	Each DBL combination exhibited synergy versus a various number of isolates, ranging from 14% (e.g. cefditoren/biapenem) and 100% (e.g. cefoxitin/imipenem). 7/13 Drug pairs exhibited synergy versus >50% of the isolates (e.g. cefuroxime/imipenem).
Kumar *et al.*, 2017^42^	*In vitro* synergy AST by chequerboard titration assay (72 h incubation at 30°C).	ATCC 19977, 4 clinical respiratory isolates from CF patients	Combinations of a carbapenem/penem^a^ (doripenem, biapenem, tebipenem, ertapenem, T205^b^, T210^b^ and faropenem) and a cephalosporin (cefdinir)	Doripenem/cefdinir showed synergy versus ATCC 19977 (FICI 0.50) and mild synergy versus the 4 clinical isolates (FICI <1).
Lefebvre *et al.*, 2016^41^	*In vitro* activity by time–kill assay (4 day incubation at 30°C). Intracellular activity in THP-1 macrophages (4 day incubation at 30°C). Impact of deletion of *bla*_Mab_ on β-lactam activity.	ATCC 19977, ΔBla_Mab_ mutant	Cefoxitin/ceftaroline, imipenem/ceftaroline, amoxicillin/ceftaroline, amoxicillin/cefoxitin, amoxicillin/imipenem, cefoxitin/imipenem	In the time–kill assay, cefoxitin/imipenem did not show superior killing activity to either β-lactam alone versus ATCC 19977, but it was significantly more active than either β-lactam alone versus ΔBla_Mab._ In the TPH-1 macrophage assay, imipenem (8 mg/L) and cefoxitin (32 mg/L) was the only combination to show improved killing activity over either drug alone versus both the strains.
Pandey *et al.*, 2019^53^	*In vitro* synergy AST by broth microdilution assay (48 h incubation at 37°C). *In vitro* activity by time–kill assay (7 day incubation at 37°C) and intracellular activity in THP-1 macrophages (24 h incubation at 37°C). *In vitro* and intracellular determination of *bla*_Mab_ expression.	ATCC 19977, 29 clinical respiratory isolates	Ceftazidime/avibactam, ceftaroline/avibactam, ceftaroline/ceftazidime, ceftaroline/ceftazidime/avibactam, imipenem/avibactam, imipenem/ceftazidime, imipenem/ceftazidime/avibactam	Ceftazidime/avibactam lowered ceftaroline and imipenem MIC_50_s by 4-fold compared with the addition of avibactam alone, versus 30 *M. abscessus* strains In time–kill assays versus ATCC 19977, the addition of ceftazidime improved the bactericidal activity of both imipenem and ceftaroline. In the macrophage assay versus 3 selected *M. abscessus* strains, ceftazidime/ceftaroline or ceftazidime/imipenem significantly reduced the cfu counts. The further addition of avibactam provided limited advantage. *bla*_Mab_ expression is enhanced in infected macrophages and in the presence of ceftaroline/ceftazidime, suggesting Bla_Mab_ may play a role in cell-wall remodelling under stressful conditions.
Guo *et al.*, 2020^56^	*In vitro* synergy AST by broth microdilution assay (48 h incubation at 37°C).	ATCC 19977, 129 clinical respiratory isolates	Ceftazidime/avibactam, imipenem/avibactam, imipenem/ceftazidime, imipenem/ceftazidime/avibactam	The addition of ceftazidime to imipenem led to a 4-fold reduction in MIC_50_s and MIC_90_s compared with imipenem alone. No significant changes in MICs were observed when imipenem was combined with avibactam.
Lopeman *et al.*, 2020^54,55^	*In vitro* synergy AST by broth microdilution assay (3–5 day incubation at 30°C).	ATCC 19977, 16 clinical isolates	Amoxicillin/relebactam, imipenem/relebactam, imipenem/amoxicillin/relebactam, ceftazidime/avibactam, ceftazidime/avibactam/amoxicillin	Imipenem/relebactam susceptibility and inhibitory activity were enhanced by the addition of amoxicillin. The inhibitory activity of ceftazidime/avibactam was increased by amoxicillin, but the concentrations of ceftazidime/avibactam were higher than those attainable in humans.
Dousa *et al.*, 2020^38^	*In vitro* synergy AST by broth microdilution assay (48 h incubation at 30°C). Molecular simulation and docking of imipenem and ceftaroline in the active site of Ldt_Mab2._	ATCC 19977, 55 clinical isolates	Imipenem/ceftaroline, imipenem/ceftaroline/relebactam	Ceftaroline/imipenem showed synergism (FICI: 0.28). Relebactam did not improve the MICs of imipenem/ceftaroline. The preferential binding of imipenem to the active site of Ldt_Mab2_ may trigger a conformational change in the enzyme, favouring the subsequent binding of ceftaroline (ligand-induced conformational change hypothesis).
Dousa *et al.*, 2022^40^	*In vitro* synergy AST by broth microdilution assay (48 h incubation at 30°C). Timed electrospray ionization MS.	ATCC 19977, 100 clinical respiratory isolates	Imipenem/amoxicillin, amoxicillin/durlobactam, imipenem/durlobactam, imipenem/durlobactam/amoxicillin, cefuroxime/amoxicillin, cefuroxime/durlobactam, cefuroxime/durlobactam/amoxicillin	Durlobactam significantly enhanced the susceptibility of *M. abscessus* isolates to amoxicillin, imipenem and cefuroxime. The triple combinations cefuroxime/durlobactam/amoxicillin and imipenem/durlobactam/amoxicillin were the most potent, leading to an MIC reduction into the submicromolar range. Durlobactam showed potent intrinsic antibacterial activity, which is confirmed by its ability to form stable complexes with Ldt_Mab2_, Ldt_Mab4_ and DDC.
Story-Roller *et al.*, 2019^52^	cfu in lung, spleen and liver tissues cultured from C3HeB/FeJ mice with aerosol-induced pulmonary infections (5 day incubation at 37°C).	ATCC 19977	5 drug combinations: imipenem/cefoxitin, imipenem/cefdinir, imipenem/doripenem, imipenem/biapenem, biapenem/avibactam	All the combinations reduced the average lung bacterial burden by at least 6 log_10_ compared with untreated controls after 4 weeks of treatment. Single-drug regimens (imipenem, doripenem, biapenem, cefdinir) were less effective than DBL regimens in reducing lung cfu count. In DBL regimens, each β-lactam was used at half the dose that was used in each single-drug group. Liver and splenic dissemination was observed in untreated mice and single-drug treatment groups, but not in DBL groups.

AST, antibiotic susceptibility testing; CF, cystic fibrosis.

^a^Faropenem.

^b^Experimental carbapenems.

Story-Roller *et al*.^31^ tested 206 antimicrobial combinations against the reference *M. abscessus* strain ATCC 19977 for synergy; pairs of two β-lactams or a β-lactam with either a rifamycin or a BLI (avibactam or clavulanate) were tested. Twenty-four combinations showed synergy. Furthermore, the MICs resulting from the observed synergisms were predicted based on the FICIs: 12 combinations—11 pairs of two β-lactams and 1 pair of a β-lactam plus avibactam—exhibited improved MICs, all within the therapeutic range. Among these, five combinations had MICs within the range of full susceptibility, while seven had intermediate susceptibility. The remaining 12 combinations displayed MICs within the range of resistance despite the synergy detected by the chequerboard assay.

Of note, the five most synergistic combinations (biapenem/avibactam, cefoxitin/imipenem, imipenem/doripenem, cefdinir/imipenem and imipenem/biapenem) comprised drugs with the lowest initial MICs when tested individually. Among carbapenems, imipenem (MIC of 8 mg/L) demonstrated synergy with nearly all the other drugs, followed by biapenem and doripenem (MICs of 16 mg/L). Among cephalosporins, cefoxitin and cefdinir showed the lowest initial MICs (64 mg/L). In line with these findings, in a further analysis by the same authors,^32^ cefoxitin/imipenem was the only combination, among 13 tested, that showed synergy against 100% of the 21 clinical isolates of *M. abscessus* examined; this was despite the large heterogeneity in genomes and drug susceptibility among the isolates. The corresponding MICs fell within the therapeutic range for all the isolates except one. On the contrary, in a previous study assessing the bactericidal activity of the same combination (cefoxitin/imipenem) against both ATCC 19977 and its β-lactamase-deficient derivative (ΔBla_Mab_),^41^ no difference in killing activity was observed between the combination and the single drugs against the parental strain. Nevertheless, the combination showed greater killing activity than the single drugs against the ΔBla_Mab_, even if with limited difference (0.5 log_10_).

#### Impact of BLI addition in DBL combinations

Bla_Mab_, a chromosomally encoded *M. abscessus* β-lactamase, efficaciously impairs the activity of a wide range of β-lactams by heightening their MICs and resulting in intrinsic resistance.^27,59^ This, along with the fact that 5 out of 24 synergistic combinations identified by Story-Roller *et al.* included avibactam,^31^ may suggest that β-lactamase inhibition could potentiate DBL efficacy against *M. abscessus*. Certain BLIs, such as avibactam, relebactam and vaborbactam, act as potent, competitive reversible inhibitors of Bla_Mab_.^38,47,54,55,60^ Previous studies showed that these inhibitors can restore and/or enhance the activity of several β-lactams, especially penicillins and cephalosporins, but also carbapenems, by reducing their MICs for *M. abscessus* in both *in vitro* and *in vivo* models.^38,41,46,47,53,60–67^ This has led to better investigation of their use within DBL strategies for *M. abscessus*, mainly focusing on the currently licensed co-formulations imipenem/relebactam and ceftazidime/avibactam. Specifically, ceftazidime/avibactam has been tested in combination with amoxicillin, ceftaroline or imipenem against *M. abscessus*, and with the similar purpose and rationale imipenem/relebactam has also been investigated in combination with ceftaroline or amoxicillin. To our knowledge, there are no studies on the other marketed co-formulation, meropenem/vaborbactam, despite the proven efficacy of vaborbactam in improving certain single β-lactam activity against *M. abscessus.*^66^

Going into more detail, ceftaroline, a broad-spectrum cephalosporin with activity against MRSA, was evaluated in combination with ceftazidime, with or without avibactam, against 30 strains, including ATCC 19977.^53^ Avibactam enhanced ceftaroline activity against *M. abscessus*, resulting in a 16-fold decrease in its MIC_50_, from 16 to 1 mg/L. The addition of ceftazidime/avibactam to ceftaroline led to an additional 4-fold decrease in MIC, compared with that of ceftaroline/avibactam alone, from 1 to 0.25 mg/L. Interestingly, the same MIC_50_ of 0.25 mg/L was obtained by combining ceftaroline with ceftazidime alone. No significant differences were seen in ceftaroline MICs when tested with either ceftazidime alone or in combination with ceftazidime/avibactam.

A similar effect was observed when combining ceftazidime with imipenem against the same 30 strains^53^ and, in a different study, against 129 clinical isolates.^56^ Unlike ceftaroline, pairing imipenem with avibactam did not result in a significant reduction in imipenem MICs. Conversely, the addition of ceftazidime/avibactam to imipenem led to a 4-fold decrease in MICs, as well as the adjunct of ceftazidime alone that also resulted in the same 4-fold MIC decrease, allowing the resistance to imipenem to be overcome. These findings suggest that for both ceftazidime/imipenem and ceftazidime/ceftaroline, the synergistic effect is mostly driven by the DBL combination rather than the BLI. Despite the scarce activity of ceftazidime alone against *M. abscessus*,^47,59^ this β-lactam in combination with imipenem or ceftaroline showed synergistic properties, with no greater effect exerted by adding a BLI (avibactam).

As for amoxicillin, prior studies demonstrated that both relebactam and avibactam can potentiate amoxicillin activity *in vitro*.^46,47,54,55,60,62^ Lopeman *et al.*^54,55^ explored the potential of combining amoxicillin with ceftazidime/avibactam against *M. abscessus*. Although amoxicillin was found to enhance the inhibitory activity of this combination, this effect was reached only for relatively high concentrations of ceftazidime/avibactam, which are not deemed suitable for clinical use.

The triple combination ceftaroline/imipenem/relebactam was tested against 55 clinical isolates and the reference strain.^38^ Ceftaroline/imipenem displayed synergism with an FICI of 0.28. The addition of ceftaroline reduced the MIC_50_ and MIC_90_ of imipenem by two dilutions or more, while the addition of relebactam to ceftaroline/imipenem did not further improve the DBL efficacy. Ceftaroline/imipenem was an effective and potent combination per se, regardless of relebactam, consistently with what has been observed for avibactam with ceftaroline/ceftazidime and ceftazidime/imipenem.

Regarding amoxicillin, growth curves were performed with ATCC 19977 in the presence of fixed doses of imipenem/relebactam (0.5–0.25 mg/L, respectively).^54,55^ Without amoxicillin, imipenem/relebactam led to growth comparable to that of the *M. abscessus* control cultures, while bacterial growth was fully inhibited afterthe addition of amoxicillin (32 mg/L). To validate this observation, *in vitro* susceptibility testing was conducted on 16 clinical isolates, all susceptible or intermediate to imipenem, and all but one resistant to both amoxicillin and relebactam. When testing imipenem/relebactam, the MICs of imipenem displayed a 2-fold reduction in almost half of the isolates, remained unchanged in 31.2% and surprisingly increased by 2-fold in 6.2% of isolates. Conversely, the addition of amoxicillin to imipenem/relebactam resulted in a significant decrease of imipenem MIC for about 94% of the isolates, with the remaining showing no variation. These data suggest that amoxicillin/relebactam potentiates imipenem activity, with greater efficacy than relebactam alone. Besides, the addition of amoxicillin allowed a 4-fold reduction in the required concentrations of imipenem/relebactam, thus minimizing unnecessary exposure to antibiotics. Of note, the combination imipenem/relebactam/amoxicillin exerted a much greater additive effect than ceftazidime/avibactam/amoxicillin. The weaker activity of ceftazidime against *M. abscessus* compared with imipenem could explain this difference.^47,59^

In a future perspective, the potential of durlobactam, a new and more potent Bla_Mab_ inhibitor, has been recently explored.^40^ The addition of durlobactam to amoxicillin/imipenem resulted in a greater reduction in MICs, compared with the one achieved by combining amoxicillin with imipenem alone. This suggests that durlobactam may act by shielding amoxicillin from Bla_Mab_ hydrolysis, thereby enabling amoxicillin to synergize with imipenem. Notably, durlobactam exhibits intrinsic antibacterial activity on its own (MIC range: 2–8 mg/L). This is due to its ability to create stable complexes with Ldt_Mab2_, Ldt_Mab4_ and DDC, while also functioning as an inhibitor of Bla_Mab_.

#### Intracellular activity

Like *M. tuberculosis*, *M. abscessus* is able to resist intracellular destruction, once it is phagocytosed by macrophages and other immune cells after tissue invasion.^14^ This ability to survive within cells and persist in the granuloma are not only key pathogenic factors, but may also influence the response to treatments.^68,69^ Research on *M. tuberculosis* demonstrated the differential susceptibility to antitubercular drugs tested at the intracellular versus extracellular levels.^70^ Consequently, data from studies that assess drug efficacy at the extracellular level may translate poorly into *in vivo* models where intracellular and extracellular phenomena take place. While this aspect has been extensively investigated in the case of the best-known *M. tuberculosis*, data are very limited for NTM. A critical step for the intracellular activity of antibiotics is the drug penetration within phagocytes.^71^ Several β-lactams possess poor penetration within the intracellular compartment, though with a certain degree of intermolecule variability;^72–74^ for example, comparing cefotaxime and imipenem by phagocyte penetration, the former possesses poor penetration, while the latter has better penetration ability, with further variability in the dynamics of different steps of intracellular entry and accumulation over time.^72^ Specifically for *M. abscessus*, few synergistic DBL combinations have also been examined through macrophage assay.

Cefoxitin/imipenem showed moderate synergism in a macrophage model, both against a WT strain and ΔBla_Mab_.^41^ However, this effect was limited to the combination of low imipenem concentrations (8 mg/L) with high cefoxitin concentration (32 mg/L). Likewise, intramacrophage activity of imipenem/ceftazidime and ceftaroline/ceftazidime was assessed against three clinical isolates.^53^ In line with the synergy demonstrated in *in vitro* assessments, both combinations drastically lowered the cfu counts, demonstrating greater intracellular activity than the single drugs alone, regardless of avibactam addition.

Lastly, Bla_Mab_ is overexpressed in macrophages infected with *M. abscessus*, contrasting with its expression in planktonic cultures.^53,64^ This raises the substantial risk of underestimating the impact of Bla_Mab_ through *in vitro* susceptibility testing, which emphasizes the need for more *in vivo* and intracellular studies. Additionally, these findings could explain why BLIs showed greater efficacy in enhancing antibiotic activity in several studies at the intracellular level rather than *in vitro* or in broth cultures.^41,60,64^ Of further concern, both in macrophages and in cellular broth cultures, an up-regulation of this β-lactamase was reported after exposure to ceftaroline/ceftazidime.^53^ On the contrary, no significant up-regulation was seen in broth cultures in the presence of imipenem. This may suggest a potential inducible mechanism of resistance under treatment, triggered by specific drugs or combinations, and a better understanding of the complex Bla_Mab_ gene regulation could inform and guide tailored therapeutic strategies.

### In vivo studies

To the best of our knowledge, only one study has assessed the efficacy of DBL therapy against *M. abscessus in vivo.*^52^ Several animal models of *M. abscessus* infection had been previously established by using larvae of zebrafish,^47,64^  *Drosophila*,^75,76^  *Galleria mellonella* and mice infected via tail vein injections,^2,77–80^ but they failed to reproduce the complex pathophysiology of invasive *M. abscessus* lung disease. For this purpose a new murine model was developed by delivering *M. abscessus* via the aerosol route in C3HeB/FeJ mice with induced immunosuppression.^52^ This model was then used to test the *in vivo* efficacy of one β-lactam/β-lactamase (biapenem/avibactam) and four DBL combinations (imipenem/cefoxitin, imipenem/cefdinir, imipenem/doripenem and imipenem/biapenem) that previously displayed *in vitro* synergism. The bacterial growth in the lungs of mice belonging to the DBL treatment groups was compared with that of the untreated and of the single-β-lactam-treated groups. In the DBL groups, the antibiotic doses were half of those used in each single-β-lactam group. After 4 weeks, all the combinations proved to be effective in reducing the mean cfu count by 6 log_10_ or more, with significant differences observed between untreated mice and each of the combination treatment groups. Single-drug regimens appeared to be less effective and bactericidal than DBL regimens. Moreover, both in untreated mice and in the single-drug-treatment groups, liver and splenic dissemination was detected, which did not occur in any of the DBL groups.

### Conclusions and future perspective

Current treatments for *M. abscessus* are prolonged and often result in high rates of failure and recurrence, which underscores the urgent need for novel therapeutic strategies. In this scenario, DBL emerges as a potential, yet underexplored resource against this mycobacterium. The rationale of considering DBL is to improve treatment outcomes by exploiting the synergism between these molecules. Several β-lactam combinations showed synergistic efficacy against both the reference strain and various clinical strains of *M. abscessus* in both *in vitro* and cell models.^31,38,53,54^ To date, promising findings have been provided for at least cefoxitin/imipenem, ceftaroline/imipenem, ceftazidime/ceftaroline and ceftazidime/imipenem, but only the cefoxitin/imipenem combination is included in the current guidelines.^19^ Overall, BLIs seem to add little or no activity to DBL combinations, although results obtained with a novel BLI, durlobactam, are encouraging.^40^ However, the synergism demonstrated *in vitro* may not necessarily correlate with clinical response and, so far, the use of DBL for *M. abscessus* in humans is limited to anecdotal reports.^81^ Further preclinical and clinical studies are warranted to better detail pharmacological data in complex systems and to optimize models for in-human generalizability. In this regard, and of concern, none of the reference studies could comprehensively take into account the effects of *in vivo* factors that influence treatment outcomes, such as biofilm formation.^10^ A thorough evaluation of pharmacokinetic/pharmacodynamic properties, determination of optimal doses, and assessment of safety profiles for these combinations is also urgently needed.
